# Iron arsenides with three-dimensional FeAs layer networks: Ca_*n*(*n*+1)/2_(Fe_1−*x*_Pt_*x*_)_(2+3*n*)_Pt_*n*(*n*−1)/2_As_(*n*+1)(*n*+2)/2_ (*n* = 2, 3)

**DOI:** 10.1038/srep39280

**Published:** 2016-12-20

**Authors:** Naoyuki Katayama, Seiichiro Onari, Kazuyuki Matsubayashi, Yoshiya Uwatoko, Hiroshi Sawa

**Affiliations:** 1 Department of Applied Physics, Nagoya University, Nagoya 464-8603, Japan; 2 Department of Physics, Okayama University, Okayama 700-8530, Japan; 3 Department of Engineering Science, University of Electro-Communications, Chofu, Tokyo 182-8585, Japan; 4 Institute for Solid State Physics, University of Tokyo, Kashiwanoha 5-1-5, Kashiwa, Chiba 277-8581, Japan

## Abstract

We report the comprehensive studies between synchrotron X-ray diffraction, electrical resistivity and magnetic susceptibility experiments for the iron arsenides Ca_*n*(*n*+1)/2_(Fe_1−*x*_Pt_*x*_)_(2+3*n*)_Pt_*n*(*n*−1)/2_As_(*n*+1)(*n*+2)/2_ for *n* = 2 and 3. Both structures crystallize in the monoclinic space group *P*2_1_/*m* (#11) with three-dimensional FeAs structures. The horizontal FeAs layers are bridged by inclined FeAs planes through edge-sharing FeAs5 square pyramids, resulting in triangular tunneling structures rather than the simple layered structures found in conventional iron arsenides. *n* = 3 system shows a sign of superconductivity with a small volume fraction. Our first-principles calculations of these systems clearly indicate that the Fermi surfaces originate from strong Fe-3*d* characters and the three-dimensional nature of the electric structures for both systems, thus offering the playgrounds to study the effects of dimensionality on high *T*_*c*_ superconductivity.

Both copper- and iron-based superconductors share structural low dimensionality, leading to the speculation that reduced dimensionality might be essential for iron-based superconductors as well as copper-oxide superconductors^1^,^2^,^3^,^4^. In fact, enhanced spin fluctuations that arise from reduced dimensionality have been regarded as a crucial factor for the emergence of high *T*_*c*_ superconductivity in iron-based systems^3^. On the other hand, some experimental results lead us to question the importance of low dimensionality for some iron-based systems: (i) nearly isotropic nature in underlying electric states^5^, (ii) fairly substantial interlayer magnetic interactions^6^,^7^,^8^,^9^,^10^,^11^, and (iii) different dimensionality of the Fermi surfaces between hole- and electron-doped systems^12^,^13^,^14^,^15^. To understand the effects of dimensionality on iron-based superconductors, the exploration of novel iron arsenides without two-dimensional lattice structures has been vigorously pursued^16^,^17^. Very recently, the emergence of pressure-induced superconductivity was reported in BaFe_2_S_3_ with quasi one-dimensional ladders, implying the unnecessarily of the structural two-dimensionality for superconductivity^18^. We expect these studies will provide important insights by comparing their physical properties and electric structures with those in conventional iron-based systems with two-dimensional structures in lattice structures. For example, spin ladders in copper oxides can be used to elucidate the high *T*_*c*_ mechanism^19^,^20^.

In this paper, we report the relationship between crystal structures and physical properties for the iron arsenides with three-dimensional FeAs networks, Ca_*n*(*n*+1)/2_(Fe_1−*x*_Pt_*x*_)_(2+3*n*)_Pt_*n*(*n*−1)/2_As_(*n*+1)(*n*+2)/2_ with *n* = 2 and 3, where the horizontal FeAs layers are bridged by inclined FeAs planes through edge-sharing FeAs_5_ square pyramids. Although the initial structural study has been already performed by another group^21^, we present the details of the crystal structures of these systems, clarified by synchrotron single-crystal X-ray diffraction experiments. We further exhibit the resistivity and magnetic susceptibility of these systems and show a sign of superconductivity in *n* = 3 systems with a small volume fraction, indicating local superconductivity in the crystal. Based on the structural parameters collected using synchrotron X-ray diffraction experiments, we clarify the three-dimensional nature in electric structures for these systems by first-principles calculations using the WIEN2k package.

## Results

Single-crystal X-ray diffraction experiments revealed that the both systems crystallize in monoclinic structures. Through careful investigation of the extinction rule of *k* = 2*n* + 1 for 0*k*0, we found that the non-centrosymmetric space group *P*2_1_ (#4) or the centrosymmetric space group *P*2_1_/*m* (#11) can be realized for both systems. Our structural analysis revealed that reliability factors based on the structural factors remain almost unchanged, regardless of the presence of centrosymmetry. Thus, we determined the space group to be *P*2_1_/*m* (#11) with higher symmetry for both systems. The refined conditions and obtained lattice parameters are summarized in Tables S1 and S2, respectively. The refined structures are stable in the temperature range between 42 K and 300 K. The temperature dependence of the lattice parameters for the *n* = 3 system is presented in Fig. S1.

**Figure 1:**
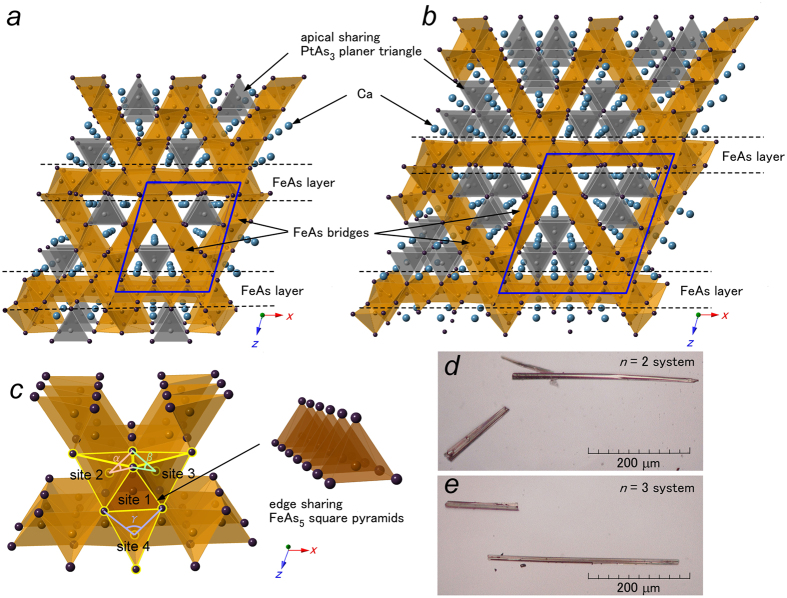
Crystal structures of the (**a**) *n* = 2 and (**b**) *n* = 3 systems with monoclinic structures [space group *P*2_1_/*m* (#11)]. The thick solid lines represent the unit cell. The FeAs layers are bridged by FeAs planes tilted at almost 60°. The FeAs planes are connected through edge sharing FeAs_5_ square pyramids. Platinum is included in all iron sites except for the sites consisting of FeAs_5_ square pyramids. (**c**) Shows the local structure around FeAs_5_ square pyramids. The FeAs_4_ tetrahedra next to the FeAs_5_ square pyramid, highlighted in yellow, are strongly distorted from the regular tetrahedron with As-Fe-As bond angle of 109.40°. The iron sites around the FeAs_5_ square pyramids are assigned as site 1–4 for discussion. See text for more detail. (**d** and **e**) Display photographs of the whisker-shaped single crystals for the *n* = 2 and 3 systems, respectively. Samples grow along the *b*-axis for both systems.

The obtained crystal structures are shown in Fig. 1a and b for the *n* = 2 and 3 systems, respectively (see the Supporting Information for crystallographic data). In both systems, *α*-type is realized, as shown in the previous study^21^. The overall appearance shows triangular tunneling structures rather than the layered structures found in conventional iron-based superconductors because of the presence of FeAs bridges connecting adjacent FeAs layers. By using the single crystal synchrotron X-ray diffraction data collected at SPring-8, we clarified that the platinum substitutions occur at all iron sites composing the FeAs layers and bridges. Further amounts of substitutions occur in FeAs bridges compared with the FeAs layers both for *n* = 2 and 3 systems, as summarized in Table S2. As shown in Fig. 1c, the FeAs layers and bridges are connected through edge-sharing FeAs_5_ square pyramids, where platinum ions are excluded. In the vacant channels enclosed with FeAs planes, apical-sharing PtAs_3_ planar triangles and calcium ions are incorporated, as shown in Fig. 1a and b.

**Figure 2:**
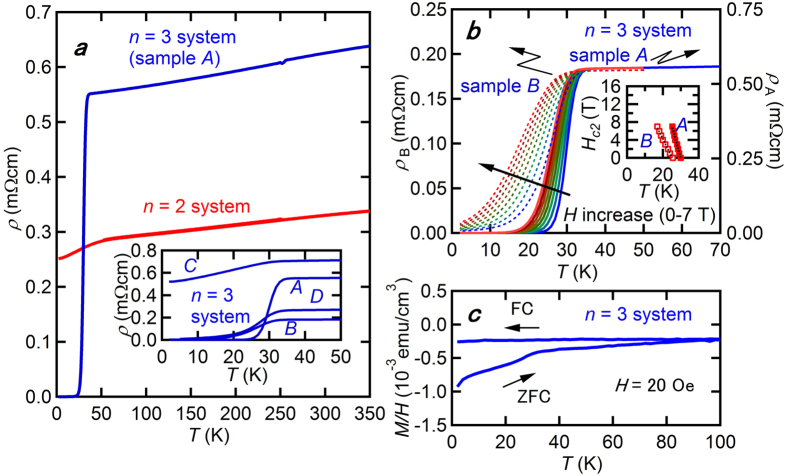
(**a**) Shows the temperature dependence of the electrical resistivity *ρ* for the *n* = 2 and 3 systems. The inset shows the low-temperature resistivity for four *n* = 3 samples, nominated by *A, B, C* and *D*. (**b**) Shows the low temperature resistivity for sample *A* and *B* in the *n* = 3 system with magnetic fields applied perpendicular to the *b*-axis up to 7 T. The magnetic field is increased at a step of 1 T. The inset shows temperature dependences of *H*_*c*2_ for sample *A* and *B*. (**c**) Shows temperature dependence of the magnetic susceptibility for the *n* = 3 system.

Our resistivity measurements show signs of a superconducting transition in the *n* = 3 system, while the *n* = 2 system is a normal metal down to 2 K. Due to the whisker-shaped single crystals that grow along the *b*-direction in relation with the tunneling structures shown in Fig. 1d and e, we can easily measure the temperature dependences of electrical resistivity along the *b*-directions for both systems. While the *n* = 2 system shows metallic conductivity without exhibiting any signs of a superconducting transition down to 2 K, the *n* = 3 systems undergo a drop in resistivity in low temperatures, which is characteristic of a superconducting transition, as presented in Fig. 2a. The onset *T*_*c*_ is 30 K, which is almost consistent among the four measured samples (samples *A*–*D*); however, the temperature dependences apparently differ depending on samples, as shown in the inset of Fig. 2a. By increasing the magnetic field perpendicular to the *b*-axis up to 7 T for sample *A* and *B*, the superconducting transition is gradually suppressed, as shown in Fig. 2b. The inset of Fig. 2b shows the temperature dependence of the upper critical field *H*_*c*2_ for sample *A* and *B*. Here, the transition temperature is determined from the midpoint of the resistive transition. For both samples, *H*_*c*2_ increases almost linearly with decreasing temperature in the 0–7 T range, although the slope differs. Corresponding to the superconducting transition in electrical resistivity, the magnetic susceptibility measurement shows a small diamagnetic signal with a shielding volume fraction (VF) of less than 1% with a small ferromagnetic hysteresis, which probably comes from the impurity phases, such as unreacted iron. Our additional magnetic susceptibility measurements using piston cylinder cell clarified that VF does not increase by applying pressure up to 1.4 GPa (data not shown): VF retains small under applied pressure.

Here, we should focus our attention on the origin of superconductivity with small VF. Although we cannot completely exclude the possibility that the tiny amount of impurities, such as Ca_10_(Pt_4_As_8_)(Fe_2−*x*_Pt_*x*_As_2_)_5_ with *T*_*c*_ = 38 K^22^, show superconductivity, we can safely say that the large amounts of impurity phases are not included in our samples based on the single crystal X-ray diffraction experimental results using the samples *A*–*D*, as shown in the Fig. S2. One possible scenario is the trace superconductivity appears in the *n* = 3 system, as is often found in another iron based compounds^23^,^24^. In the present systems, fairly amounts of platinum ions are doped to the iron sites, which may introduce the local strain originating from the inhomogeneous distribution of dopants, leading to the trace superconductivity. Considering that the bulk superconductivity often arises by the appropriate substitution^25^, further exploration of these systems may give us opportunities to study the bulk superconductivity.

For further considering the possibility for realizing superconductivity on the three-dimensional network structures, it is worth discussing the regularity of FeAs_4_ tetrahedra in the present *n* = 2 and 3 systems because the intimate relationship between the regularity of FeAs_4_ tetrahedra and the superconducting transition temperatures has already been clarified for conventional iron based superconductors with two-dimensional lattice networks^26^. In the present *n* = 2 and 3 systems, the FeAs planes and bridges are constructed by several crystallographically inequivalent FeAs_4_ tetrahedra, resulting in differing regularity depending on the site. As a common feature between the present systems, the FeAs_4_ tetrahedra around the FeAs_5_ square pyramids are strongly distorted. The As-Fe-As bond angles of *α, β* and *γ* with the arsenic ions composed of FeAs_5_ square pyramids (Fig. 1c), are much smaller than the angle (109.47°) of the regular tetrahedron; *α* = 99.774°, *β* = 102.781°, and *γ* = 95.253° are realized in the *n* = 2 system, while *α* = 102.42°, *β* = 105.296°, and *γ* = 93.236° are found in the *n* = 3 system. The regularity of the FeAs_4_ tetrahedra gradually recovers away from the FeAs_5_ square pyramids, indicating that the longer distance between neighboring FeAs_5_ square pyramids is favorable to superconductivity by increasing the more regular FeAs_4_ tetrahedra. Further explorations of homologous compounds with *n* ≥ 4 may be leading to the bulk superconductivity with three-dimensional structure.

**Figure 3:**
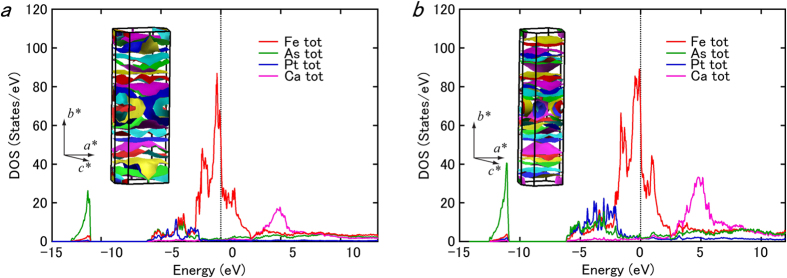
Fermi surface of the (**a**) *n* = 2 and (**b**) *n* = 3 systems calculated using the WIEN2k package. The plots show the partial DOS of the composing elements, while the insets show the overall appearance of the Fermi surfaces.

**Figure 4:**
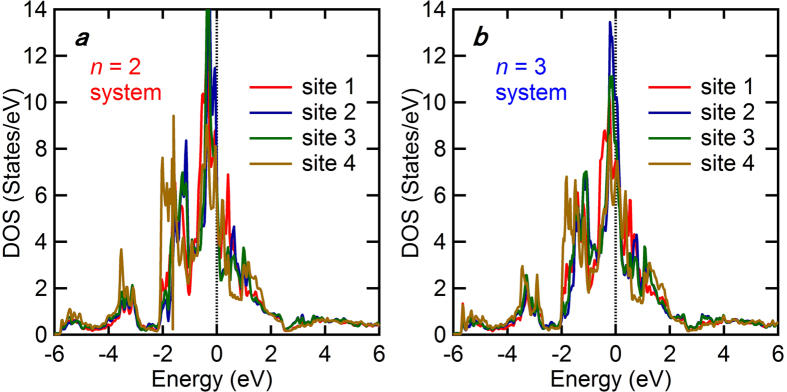
Partial DOS of the iron sites around FeAs_5_ square pyramids. Sites 1–4 are assigned in Fig. 1c. (**a** and **b**) Show the data for the *n* = 2 and 3 systems, respectively.

Finally, we would like to present the three-dimensional electric structures for the present systems clarified by the first-principles calculations using the WIEN2k package^27^ to investigate the relationship between the electric and lattice structures. For simplification, we performed the calculations for Ca_*n*(*n*+1)/2_(Fe_1−*x*_Pt_*x*_)_(2+3*n*)_Pt_*n*(*n*−1)/2_As_(*n*+1)(*n*+2)/2_ for *n* = 2 and 3 without replacing Fe with Pt, although these systems have not been experimentally synthesized. The structural parameters supplied in Table S2 were employed for the calculation. Figure 3a and b show the results of our first-principles calculations. The overall appearances of Fermi surfaces are much more complex than those in other iron-based superconductors. As shown in the insets of Fig. 3a and b, both Fermi surfaces originate from strong Fe-3*d* characters, without any dominant contributions from other elements such as Ca, As, and Pt, which is a common feature among conventional iron-based superconductors. Further investigation of the partial DOS for each Fe site confirmed that the Fermi surfaces are constructed by the contributions of all iron sites including site 1 at the FeAs_5_ square pyramid and sites 2–4 at the strongly distorted FeAs_4_ tetrahedra, as shown in Fig. 4a and b. These results clearly indicate the three-dimensional nature of the electric structures with strong Fe-3*d* characters of the present systems, which should be compared with the two-dimensional nature in the conventional iron-based superconductors.

The electrical dimensionality has been thought to be an essential factor for high *T*_*c*_ superconductivity for iron-based superconductors as well as copper-oxide superconductors. The extensive studies have been performed toward the realization of superconductivity with lower dimensionalities in electric structures^16^,^17^, inspired by the superconducting ladders in copper oxides^19^,^20^. However, the question whether the high *T*_*c*_ superconductivity appears in the compounds with the three-dimensional lattice or not has never been answered because of the lack of the proper candidate materials. Therefore, the present systems are attractive for systematic studies of the dimensional effects on iron arsenides with FeAs planes; one-dimensional systems such as those in the spin-ladder compound BaFe_2_S_3_^18^ and its derivatives^28^, two-dimensional systems in the conventional high *T*_*c*_ superconductors such as LaFeAsO, and the present three-dimensional systems, Ca_*n*(*n*+1)/2_(Fe_1−*x*_Pt_*x*_)_(2+3*n*)_Pt_*n*(*n*−1)/2_As_(*n*+1)(*n*+2)/2_. Although the bulk superconductivity has not been confirmed in the present systems, the presence of the FeAs layers and the strong Fe-3*d* characters are common features among iron based superconductors, suggesting the present homologous systems are possible parents for high *T*_*c*_ superconductivity with three-dimensional structures.

## Methods

Single crystals of the *n* = 2 and 3 systems were grown by heating a mixture of Ca, FeAs, As, Fe and Pt powders in a composition ratio of Ca/Fe/Pt/As = 6:5:13:12 and 12:11:17:20, respectively. The mixtures were placed in BN crucibles and sealed in an evacuated quartz tube. All manipulation was performed in a glove box filled with nitrogen gas. The ampules were heated at 700 °C for 24 h and then at 1000–1100 °C for 48 h, after which the samples were cooled to room temperature at a rate of 1 K/min. In relation to the structural topology of systems, whisker-shaped single crystals with typical dimensions of 1 × 0.02 × 0.02 mm^3^ were successfully obtained, which are clearly different from those in conventional iron-based superconductors with plate-like single crystals. The crystals were characterized by single-crystal X-ray diffraction using the BL02B1 beamline equipped at the SPring-8 synchrotron radiation facility (Japan); the X-ray wavelength was 0.35 Å. The temperature dependence of the crystal structures and the absence of the large amount of the impurity phase in the sample *A*–*D* were studied using the BL-8A beamline at the KEK facility (Japan); the X-ray wavelength was 0.69 Å. Powder synchrotron X-ray diffraction experiments were performed using the 11-BM beamline at the Advanced Photon Source facility (U.S.); the X-ray wavelength was 0.4138 Å. The electrical resistivity and magnetization were measured using Quantum Design PPMS and MPMS, respectively, which are equipped at the Institute for Solid State Physics (ISSP), in Japan. Magnetic susceptibility measurement of the *n* = 3 system under high pressure was performed using a piston-cylinder clamped cell with glycerin as a liquid pressure medium. The applied pressures were determined by the pressure dependence of the superconducting transition temperature of Tin.

## Additional Information

**How to cite this article**: Katayama, N. *et al*. Iron arsenides with three-dimensional FeAs layer networks: Ca_*n*(*n*+1)/2_(Fe_1−*x*_Pt_*x*_)_(2+3*n*)_Pt_*n*(*n*−1)/2_As_(*n*+1)(*n*+2)/2_ (*n* = 2, 3). *Sci. Rep.* **6**, 39280; doi: 10.1038/srep39280 (2016).

**Publisher's note:** Springer Nature remains neutral with regard to jurisdictional claims in published maps and institutional affiliations.

## Supplementary Material

Supplementary Information
