# Supplementary material for: Iron arsenides with three-dimensional FeAs layer networks: Can(n+1)/2(Fe1−xPtx)(2+3n)Ptn(n−1)/2As(n+1)(n+2)/2 (n = 2, 3)
Source: Sci Rep. 2016 Dec 20;6:39280. doi: 10.1038/srep39280 (PMC5171830; doi:10.1038/srep39280)
Supplement: Supplementary Information [file srep39280-s1.doc]

Supporting Information

Iron arsenides with three-dimensional FeAs layer networks: Ca*_n_*(*_n_*_+1)/2_(Fe_1−_*_x_*Pt*_x_*)_(2+3_*_n_*)Pt*_n_*(*_n_*_−1)/2_As(*_n_*_+1)(_*_n_*_+2)/2_ (*n* = 2, 3)

Naoyuki Katayama^1^, Seiichiro Onari^2^, Kazuyuki Matsubayashi^3^, Yoshiya Uwatoko^4^ and Hiroshi Sawa^1^

^1^Department of Applied Physics, Nagoya University, Nagoya 464-8603, Japan

^2^Department of Physics, Okayama University, Okayama 700-8530, Japan

^3^Department of Engineering Science, University of Electro-Communications, Chofu, Tokyo 182-8585, Japan

^4^ Institute for Solid State Physics, University of Tokyo, Kashiwanoha 5-1-5, Kashiwa, Chiba 277-8581, Japan

Introduction

This supporting information is provided in support of the main text with details. Table SI presents the data collections and refinements for the *n* = 2 and 3 systems. Table SII provides the crystallographic parameters for the *n* = 2 and 3 systems determined using the Synchrotron X-ray diffraction data collected at 100 K. The data were collected at SPring-8 BL02B1. Figure SI shows the temperature dependences of lattice parameters for both samples collected at KEK BL8A. Figure SII shows the absence of large amounts of impurity phase in *n* = 3 compound used for the resistivity experiments.

Table SI. Data collection and refinement statistics for the synchrotron X-ray structure determination of Ca_3_(Fe,Pt)_8_PtAs_6_ (*n* = 2 system) and Ca_6_(Pt,Fe)_11_Pt_3_As_10_ (*n* = 3 system), respectively. The data was collected at SPring-8 BL02B1 with the wave length of 0.35 Å.


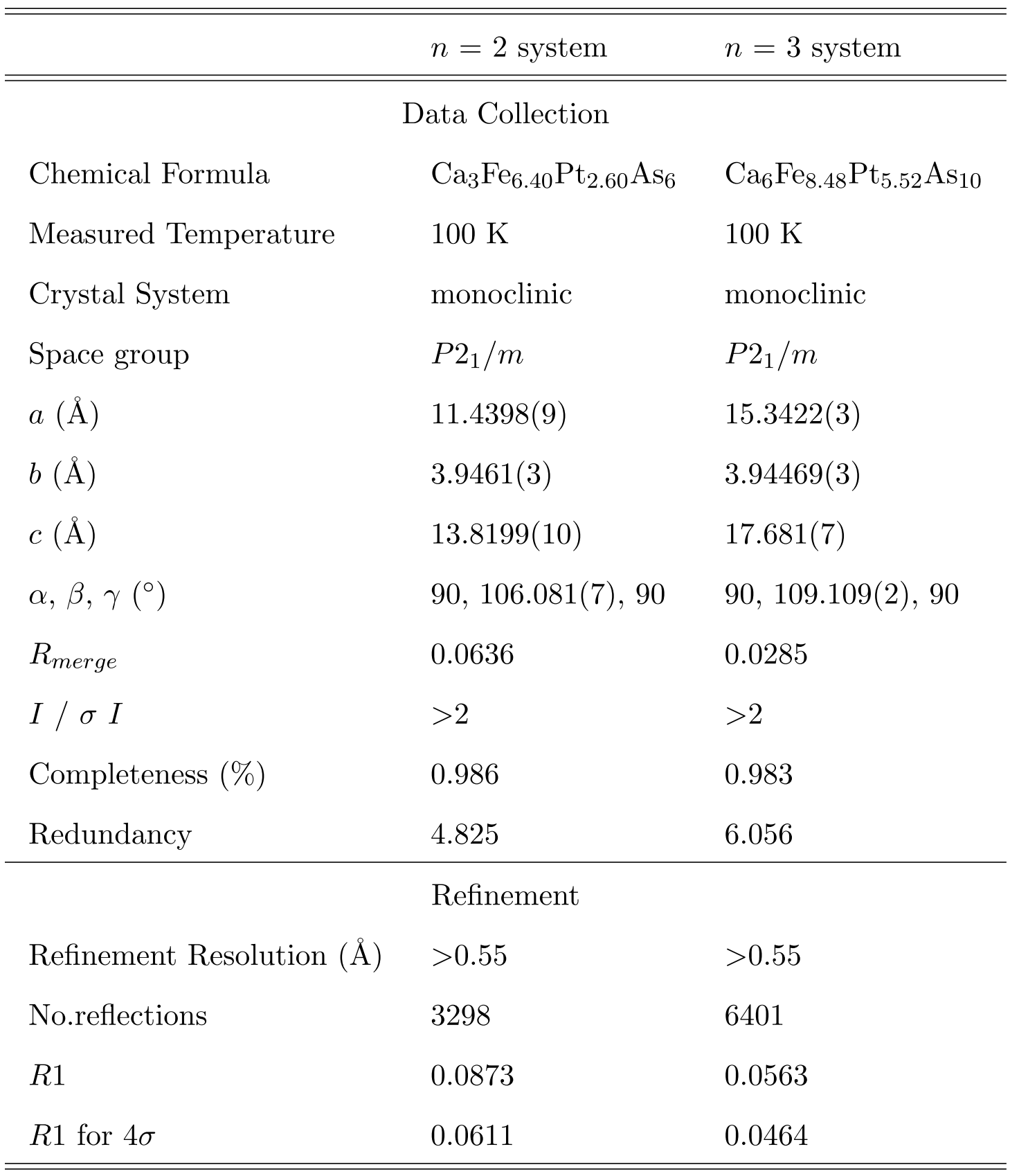


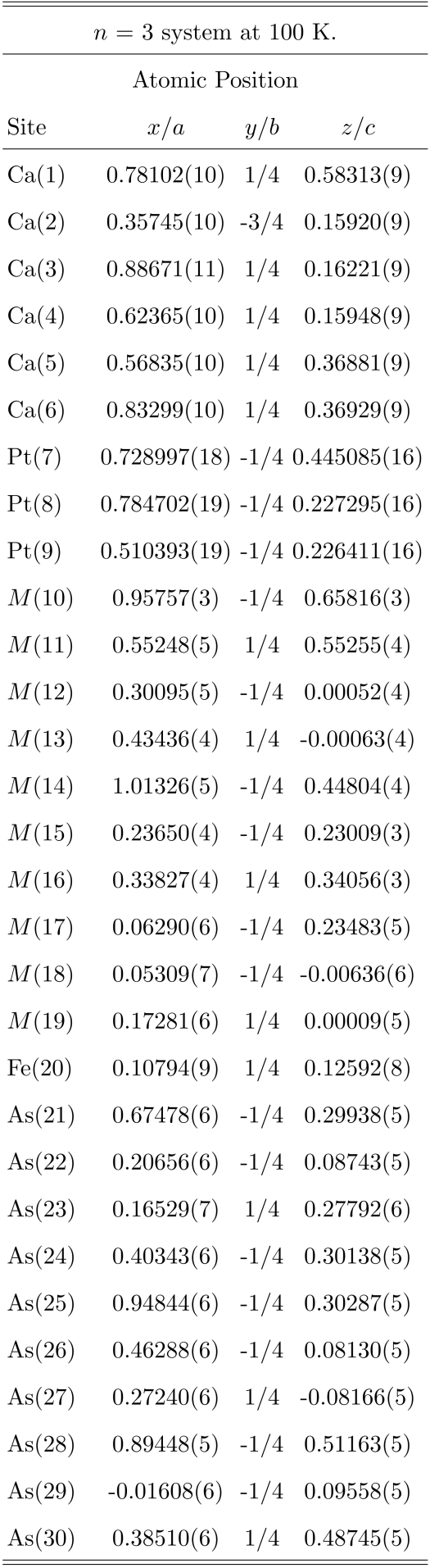

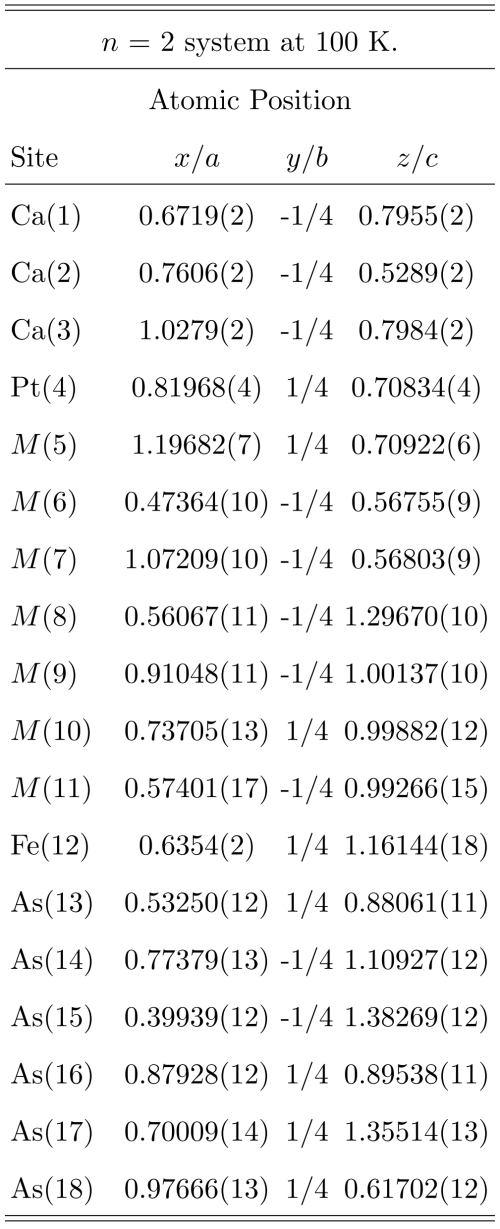
Table SII. Structural parameters collected at 100 K for the *n* = 2 and 3 systems. For the *n* = 2 system, *M*(5)-*M*(11) represent Fe_1-_*_x_*Pt*_x_*, where *x* is 0.554(5) for *M*(5), 0.265(5) for *M*(6), 0.251(5) for *M*(7), 0.250(5) for *M*(8), 0.183(5) for *M*(9), 0.097(5) for *M*(10) and 0.007(5) for *M*(11). For the *n* = 3 system, *M*(10)-*M*(19) represent Fe_1-_*_x_*Pt*_x_*, where *x* is 0.439(3) for *M*(10), 0.242(3) for *M*(11), 0.239(3) for *M*(12), 0.266(3) for *M*(13), 0.195(3) for *M*(14), 0.469(3) for *M*(15), 0.392(3) for *M*(16), 0.156(3) for *M*(17), 0.007(3) for *M*(18) and 0.119(3) for *M*(19).


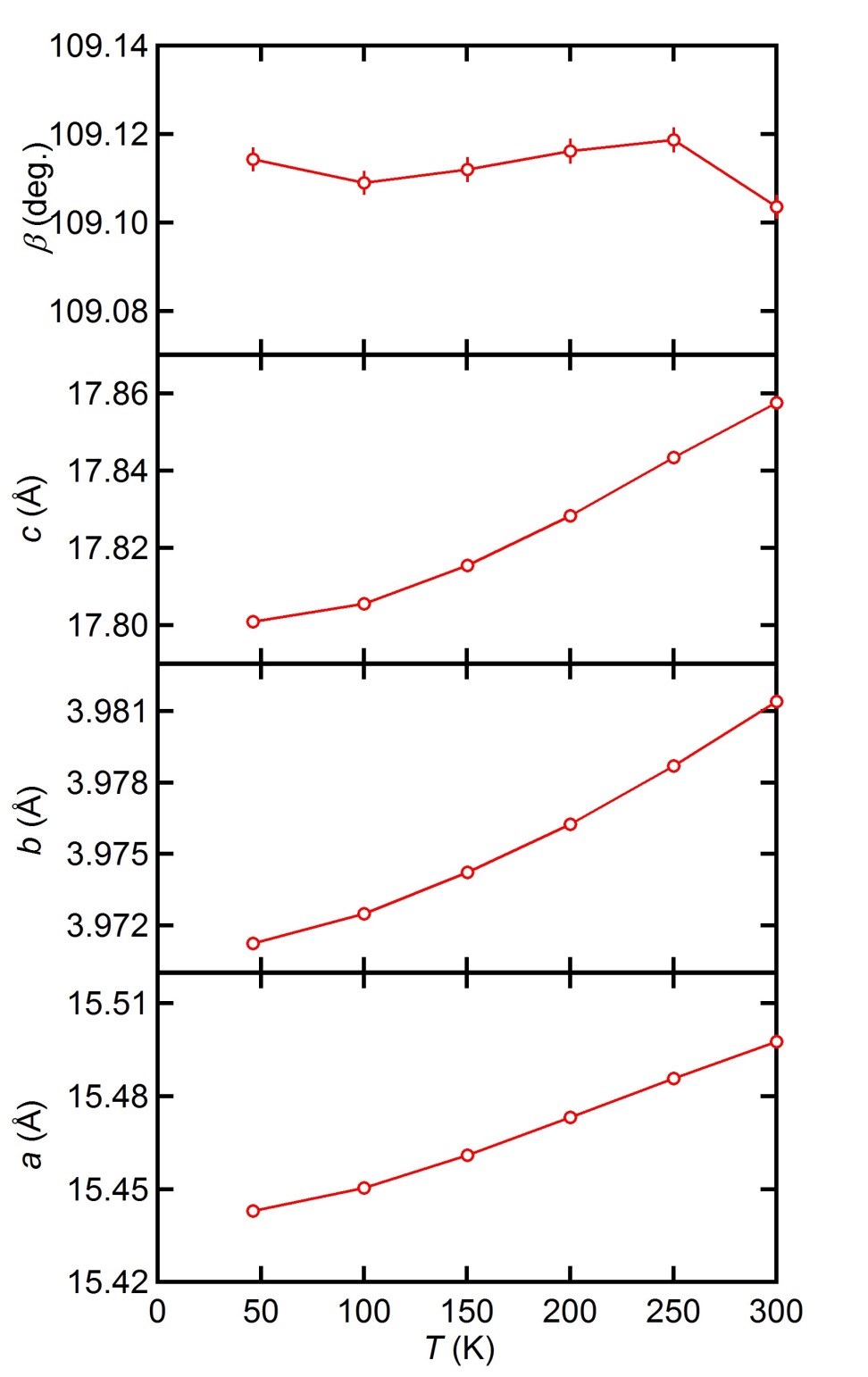


Fig SI. Temperature dependences of lattice parameters on *n* = 3 system. Red open circles indicate data collected in BL-8A equipped at the KEK facilities (Japan) using a single crystalline sample. The present parameters at 300 K are almost consistent with those obtained at 300 K in powder X-ray diffraction experiments performed in the 11-BM beamline equipped at Advanced Photon Source facility (U.S.).


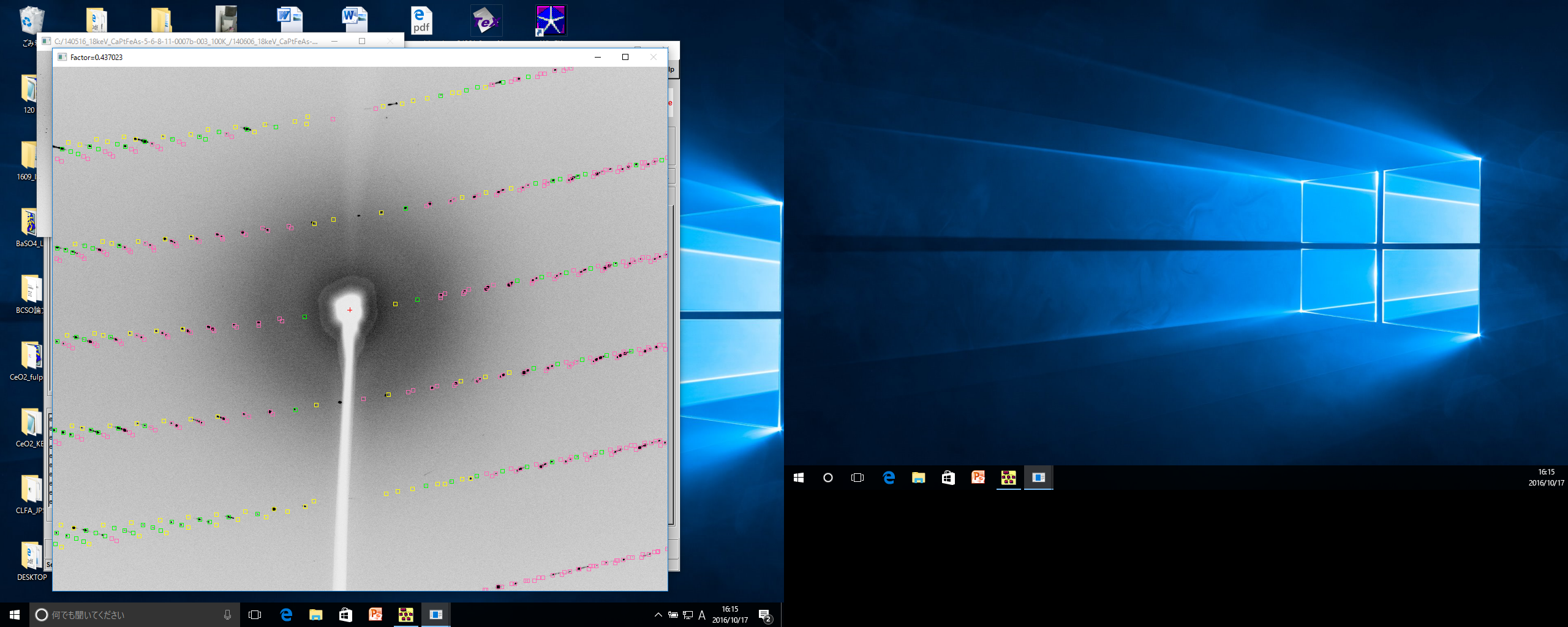


Fig SII. Synchrotron X-ray single crystal X-ray diffraction data of *n* = 3 system. The sample was used for resistivity experiment to observe trace superconductivity before X-ray diffraction experiment. Almost all Bragg peaks can be successfully indexed, indicating the absence of large amounts of impurity phase.
